# Exosomal miRNAs from Peritoneum Lavage Fluid as Potential Prognostic Biomarkers of Peritoneal Metastasis in Gastric Cancer

**DOI:** 10.1371/journal.pone.0130472

**Published:** 2015-07-24

**Authors:** Motohiko Tokuhisa, Yasushi Ichikawa, Nobuyoshi Kosaka, Takahiro Ochiya, Masakazu Yashiro, Kosei Hirakawa, Takashi Kosaka, Hirochika Makino, Hirotoshi Akiyama, Chikara Kunisaki, Itaru Endo

**Affiliations:** 1 Department of Gastroenterological Surgery, Yokohama City University Graduate School of Medicine, Yokohama, Japan; 2 Department of Clinical Oncology, Yokohama City University Graduate school of Medicine, Yokohama, Japan; 3 Division of Molecular and Cellular Medicine, National Cancer Center Research Institute, Tokyo, Japan; 4 Department of Surgical Oncology, Osaka City University Graduate School of Medicine, Osaka, Japan; University of Connecticut Health Center, UNITED STATES

## Abstract

Peritoneal metastasis is the most frequent type of recurrence in patients with gastric cancer (GC) and is associated with poor prognosis. Peritoneal lavage cytology, used to evaluate the risk of peritoneal metastasis, has low sensitivity. Here, we assessed the diagnostic potential of exosomal miRNA profiles in peritoneal fluid for the prediction of peritoneal dissemination in GC. Total RNA was extracted from exosomes isolated from six gastric malignant ascites (MA) samples, 24 peritoneal lavage fluid (PLF) samples, and culture supernatants (CM) of two human gastric carcinoma cell lines that differ in their potential for peritoneal metastasis. Expression of exosomal miRNAs was evaluated with Agilent Human miRNA microarrays and quantitative reverse transcription polymerase chain reaction (qRT-PCR). The microarray analysis indicated a low variability in the number and signal intensity of miRNAs detected among the samples. In the six MA fluids, miR-21 showed the highest signal intensity. We identified five miRNAs (miR-1225-5p, miR-320c, miR-1202, miR-1207-5p, and miR-4270) with high expression in MA samples, the PLF of serosa-invasive GC, and the CM of a highly metastatic GC cell line; these candidate miRNA species appear to be related to peritoneal dissemination. Differential expression of miR-21, miR-320c, and miR-1225-5p was validated in the PLF of serosa-invasive and non-invasive GC by qRT-PCR and miR-21 and miR-1225-5p were confirmed to be associated with serosal invasion in GC. PLF can be used to profile the expression of exosomal miRNAs. Our findings suggest that miR-21 and miR-1225-5p may serve as biomarkers of peritoneal recurrence after curative GC resection, thus providing a novel approach to early diagnosis of peritoneal dissemination of GC.

## Introduction

MicroRNAs (miRNAs) are small (19–23 nucleotides) non-coding RNAs that function as post-transcriptional regulators of gene expression and play important roles in the control of many biological processes, including cell differentiation, proliferation, and apoptosis [1]. MiRNAs have been shown to have oncogenic or tumour-suppressing activity, and deregulated expression of many miRNA species has been detected in several cancers, suggesting potential application of miRNAs as biomarkers of cancer progression and metastasis [2–5].

MiRNAs are wrapped in secretory microvesicles (e.g. exosomes, apoptotic bodies, and shedding microvesicles), which shield miRNAs from degradation by RNAse and serve as vehicles for miRNA secretion into extracellular space and body fluids. Exosomes are small membranous vesicles that have been implicated in cellular immune responses; however, recently it has become evident that exosomes contain substantial amounts of RNA and may be involved in immune-independent regulatory mechanisms. It has now been established that exosomes can perform intercellular transfer of miRNAs, thus participating in miRNA-based signalling mechanisms [6]. Higher levels of miRNA-containing exosomes have also been detected in the plasma of cancer patients than in that of healthy individuals [7], suggesting that exosome-based miRNA secretion is involved in cancer development. Analysis of aberrant miRNA expression in the serum, saliva, faeces, and urine has been employed for the early detection of B-cell lymphoma, and oral, intestinal, and bladder cancers [8–11].

Gastric cancer (GC) is the second most common cause of cancer-related death worldwide [12]. The peritoneum is the most frequent site of metastasis and recurrence of GC, and malignant ascites (MA), caused by peritoneal dissemination of cancer cells, have been associated with poor prognosis. Currently, there are no reliable predictors for the development of malignant peritoneal ascites in GC patients. MiRNA-containing exosomes represent a novel mechanism of intercellular signalling and may yield insights into the environment that permits cancer dissemination in the peritoneum [13].

In this study, exosomes were isolated from MA and the intraoperative peritoneal lavage fluid (PLF) samples obtained from GC patients, and the culture medium (CM) of highly invasive peritoneal cell lines, and miRNAs were then extracted from these samples. The quality of the extracted exosomal miRNAs and the consistency of miRNA expression patterns were verified. MiRNA expression profiles in MA, PLF, and CM samples were compared and candidate miRNAs related to peritoneal dissemination of GC were identified.

## Materials and Methods

### Patients

All patients or their guardians provided written informed consent. The study was approved by the Ethics Committee of Yokohama City University Hospital (Approval No. A110127001).

MA and intraoperative PLF samples were collected from GC patients with the clinicopathological characteristics presented in Table 1. All patients had previously been diagnosed with GC by histopathological analysis of biopsy samples taken from the primary lesions, and six MA patients had been diagnosed for the presence of ascites by abdominal computed tomography (CT) scan. Ascites were also analysed by cytology and all were confirmed as positive for malignancy by pathologists; the remaining MA samples were used for miRNA isolation. PLF was intraoperatively collected from 24 GC patients (Table 1). After laparotomy, 100 mL of normal saline was poured into the pouch of Douglas and peritoneal lavage water was collected before surgical resection of the tumour. PLF was analysed by cytology and used for miRNA isolation. Among the 24 PLF samples, six were assayed using miRNA microarray and 18 were analysed by qPCR.

**Table 1:** Clinicopathological characteristics of patients with gastric cancer. Well-differentiated adenocarcinoma (well); moderately differentiated adenocarcinoma (mod); poorly differentiated adenocarcinoma (poor). Malignant ascites (MA); Peritoneal lavage fluid (PLF); TMN stage (UICC 7th edition); Present (P) Absent (A); Peritoneal lavage cytology (CY);

Experiment	Sample	Age	Sex	T stage	N stage	M stage	TMN stage	CY	Peritoneal Dissemination	Ascites	Histological type
Microarray	MA 1	62	F	4a	3a	1	IV	+	P	P	poor
	MA 2	65	M	Resected	2	1	IV	+	P	P	mod
	MA 3	72	M	Resected	3b	1	IV	+	P	P	poor
	MA 4	71	M	4a	2	1	IV	+	P	P	poor
	MA 5	41	M	4a	2	1	IV	+	P	P	poor
	MA 6	35	F	Resected	3b	1	IV	+	P	P	poor
	PLF 1	54	M	1b	0	0	IA	-	A	A	poor
	PLF 2	67	F	4a	0	0	IIB	-	A	A	poor
	PLF 3	74	F	1b	0	0	IA	-	A	A	poor
	PLF 4	46	M	4a	0	0	IIB	-	A	A	well
	PLF 5	75	M	4a	1	1	IV	-	P	A	well
	PLF 6	55	M	4a	1	1	IV	+	A	A	poor
PCR	PLF 7	42	F	4a	1	0	IIIA	-	A	P	poor
	PLF 8	63	M	4a	1	0	IIIA	-	A	A	poor
	PLF 9	76	M	4a	2	0	IIIB	-	A	A	poor
	PLF 10	84	M	4a	3a	0	IIIC	-	A	A	mod
	PLF 11	75	M	4a	3b	1	IV	-	P	A	poor
	PLF 12	67	M	4b	1	1	IV	+	P	A	mod
	PLF 13	78	F	4a	0	0	IIB	-	A	A	poor
	PLF 14	61	M	4a	0	1	IV	-	A	A	poor
	PLF 15	62	M	4a	0	0	IIB	-	A	A	mod
	PLF 16	71	M	2	1	0	IIA	-	A	A	mod
	PLF 17	56	M	3	1	0	IIB	-	A	A	well
	PLF 18	68	M	3	1	0	IIB	-	A	A	well
	PLF 19	62	F	1	0	0	IA	-	A	A	poor
	PLF 20	63	F	1	0	0	IA	-	A	A	poor
	PLF 21	74	M	1	0	0	IA	-	A	A	well
	PLF 22	68	F	1	0	0	IA	-	A	A	poor
	PLF 23	69	M	3	0	0	IIA	-	A	A	poor
	PLF 24	88	M	1	0	0	IA	-	A	A	mod

### Cell culture

The OCUM-2M cell line was established from a primary tumour of scirrhous gastric carcinoma and the OCUM-2MD3 cell line was derived from OCUM-2M cells with a high potential for peritoneal dissemination in nude mice [14]. The cells were cultured in Dulbecco’s modified Eagle’s medium (DMEM; Life Technologies, Grand Island, NY) supplemented with 10% heat-inactivated foetal bovine serum (FBS) and antibiotic–antimycotic at 37°C in a 5% CO_2_ incubator. To obtain cell CM, OCUM-2M and OCUM-2MD3 cells were washed three times with serum-free DMEM supplemented with antibiotic–antimycotic and incubated in the same medium for 48 h.

### Isolation of exosomes

Samples for exosome isolation were prepared as described previously [15]. To remove large cell particles and cell debris, MA, PLF, and CM were centrifuged at 2,000 × *g* for 15 min at 4°C and filtered through a 0.22-μm filter (Millipore, Billerica, MA). The supernatant was stored at −80°C until required for miRNA extraction.

To precipitate exosomes, samples were centrifuged at 110,000 × *g* for 70 min at 4°C. The exosome pellet was washed with 11 mL of phosphate-buffered saline (PBS) and centrifuged again as described. The pellet was used for RNA extraction.

### Total RNA extraction and analysis

Total RNA was extracted from exosomes using an miRNeasy mini kit (Qiagen, Hilden, Germany) according to the manufacturer’s instructions and was then stored at −80°C until required further analysis.

The quality, concentration, and size of total exosomal RNA were assessed using an Agilent Bioanalyzer 2100 (Agilent Technologies, Foster City, CA). Prior to the analysis, total RNA was prepared using an Agilent RNA 6000 Pico kit (Agilent Technologies) according to the manufacturer's protocol.

### MiRNA microarray

MiRNAs extracted from the six MA, six PLF, and two CM samples were analysed using Agilent Human miRNA Microarrays (Agilent Technologies), which contain 1,226 human miRNAs and 146 human viral miRNAs. For miRNA detection, 100 ng RNA was labelled and hybridised using the Human miRNA Microarray kit (Rel 16.0; Agilent Technologies) according to the manufacturer's protocol (miRNA Complete Labeling and Hyb kit for miRNA Microarray System). Hybridisation signals were detected with a DNA Microarray Scanner G2505C (Agilent Technologies) and the scanned images were analysed using the Agilent Feature Extraction Software (v. 10.7.3.1). Data analysis was performed using Agilent GeneSpring GX software v. 11.0.2. (log_2_ transformation). Baseline transformation was not performed.

The quality of miRNA extraction was analysed according to the variability in the number of microarray-detected miRNA species, signal intensity, and the correlation of miRNA expression among samples according to mean ± SD, coefficient of variation (CV), and correlation coefficient (CC).

### Selection of peritoneal metastasis-specific miRNAs

The expression profiles of exosomal miRNAs were analysed to select miRNAs that are specific for GC peritoneal dissemination using the following step-wise approach. Step 1: miRNA profiles in the CM of the highly metastatic peritoneal OCUM-2MD3 cells were compared with those of the parental non-metastatic OCUM-2M cells, and differentially expressed miRNAs (mean fold-change, >2) were selected.

Step 2: six PLF samples were classified into two groups: T4 (n = 4) and T1–T3 (n = 2), according to cancer stage by tumour size and extension (UICC-AJCC, 7^th^ edition). Stage T4 GC is characterised by the highest frequency of peritoneal metastases as compared to other stages [16]. The miRNA expression profiles of T4 group were compared with those of the T1–T3 group and differentially expressed miRNAs (mean fold-change, >2) were selected.

Step 3: the selected miRNAs that were common to both steps and that were expressed in MA were further filtered according to signal intensity. Those with intensity values of more than log_2_ 10 in MA and PLF were selected as candidate miRNAs related to peritoneal metastases; miR-21 showed the highest average signal intensity among MA samples. The differential expression of miRNA species selected in Step 3 was validated in the remaining 18 PLF samples by qRT-PCR.

### Quantitative analysis of peritoneal metastasis-specific miRNA expression by qRT-PCR

TaqMan microRNA assays (Life Technologies) were used to quantify the relative expression levels of miR-21 (assay ID. 000397), miR-320c (assay ID. 241053_mat), miR-1225-5p (assay ID. 002764), and miR-16 (assay ID. 000391). Reverse transcription was performed using the TaqMan miRNA RT Kit (Life Technologies) under the following conditions: 30 min at 16°C, 30 min at 42°C, and 5 min at 85°C. PCR was carried out in 96-well plates using the 7500 Real Time PCR System (Life Technologies); all reactions were performed in triplicate. The expression levels of target miRNAs were normalised to that of miR-16, which was used as a stably expressed control [17]. When analysing our microarray data of exosomal miRNA in malignant ascites samples, miR-16 presented the lowest coefficient of variation (CV = 4%) among the candidate internal standards, including miR-638 (CV = 8%), let-7a (CV = 8%), and 142-3p (CV = 23%).

### Statistical analysis

Continuous variables were compared using Student’s *t*-test. When necessary, the *t*-test was modified to compare unequal variances. The difference between categorical variables was evaluated using Fisher’s exact test. Correlation between two variables was evaluated using Pearson correlation coefficient. Differences were considered statistically significant at P < 0.05. Statistical analyses were conducted using SPSS 21.0 software for Windows (IBM, Armonk, NY).

## Results

### Quantitative analysis of extracted exosomal RNA

**Fig 1:**
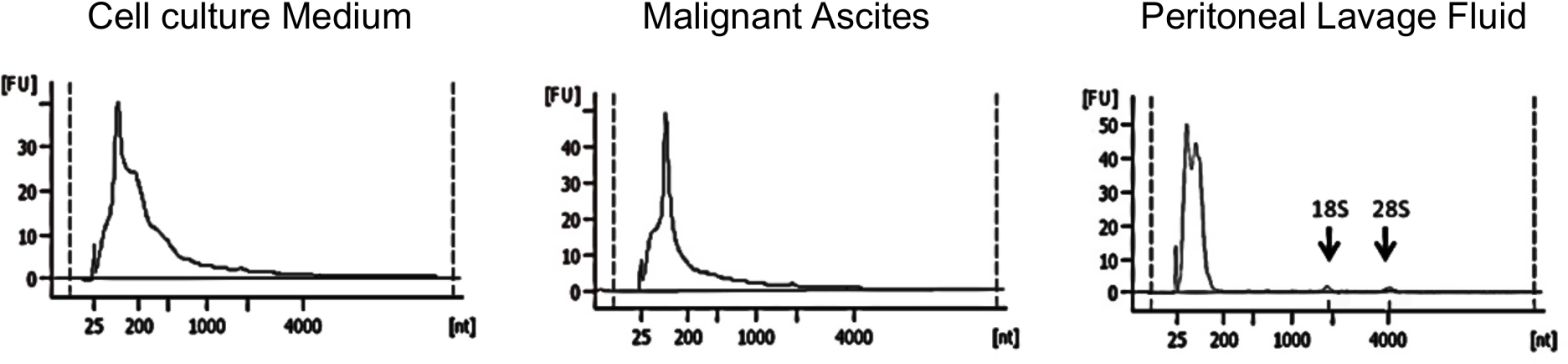
Exosomal miRNA expression in malignant ascites (MA), peritoneal lavage fluid (PLF), and cell culture media (CM). Variation and commonality between the samples were examined. Evaluation of total exosomal RNA used a Bioanalyzer 2100. The electropherograms show the size distribution (nucleotides, nt) and fluorescence intensity (FU) of total exosomal RNA isolated from CM, MA, and PLF. The lowest spike (around 25 nt) in each sample is the marker of the Agilent RNA 6000 Pico kit.

Total RNA was extracted from exosomes isolated from MA, intraoperative PLF, and cell CM. The median concentration of the extracted exosomal RNA was 4.4 ng/μL (ranging from 1.2 to 6.1 ng/μL) for MA and 6.4 ng/μL (from 1.7 to 16.4 ng/μL) for PLF. Electropherograms revealed a large proportion of small RNA species present in all CM and clinical samples, while no or very little contamination with 18S and 28S ribosomal RNA (no distinct peaks at the corresponding molecular weight) was observed (Fig 1).

### Performance of miRNA microarrays

**Fig 2:**
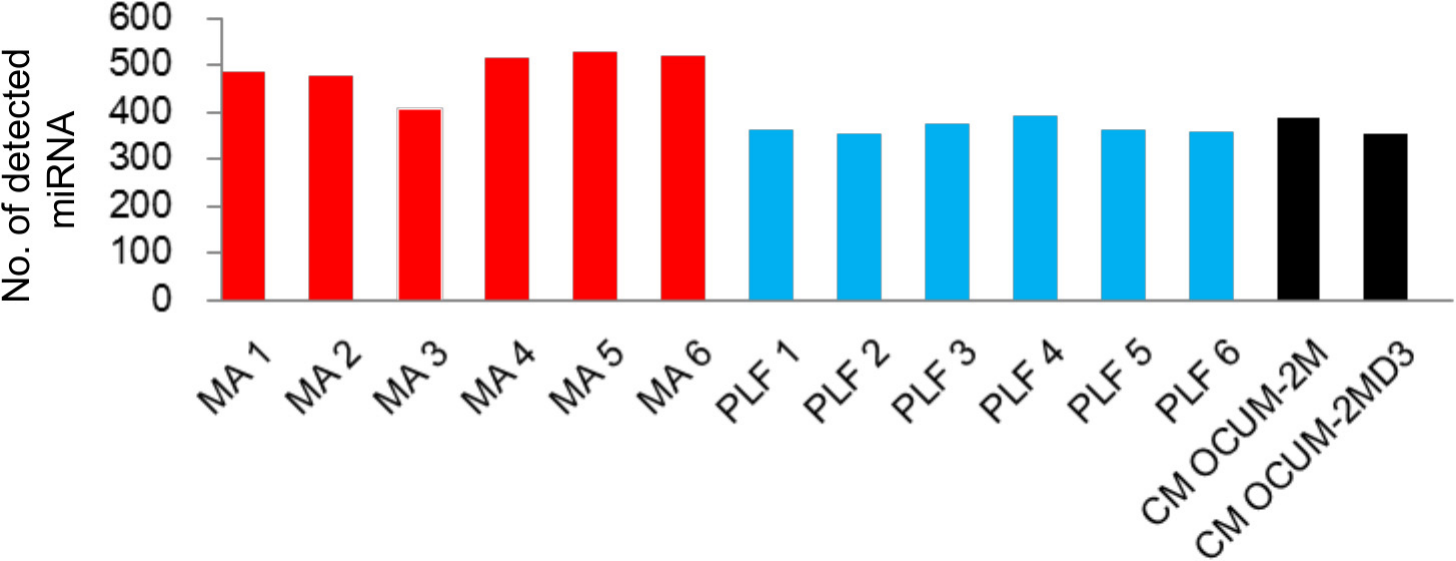
The number of detected miRNA species in 14 samples.

To test the expression of exosomal miRNA microarrays in each group of samples, we examined the number of detected miRNAs, percentile of signal intensity, the number of commonly captured miRNAs, and the correlation of miRNA expression among the groups. The numbers of miRNA species detected in each group by microarray is shown in Fig 2. In the six MA and six PLF samples, these mean numbers were 490.1 (SD = 42.3; CV = 8.6%) and 367.5 (SD = 13.4; CV = 3.6%), respectively. In each group, the variability in the number of detected miRNAs among samples was low.

**Fig 3:**
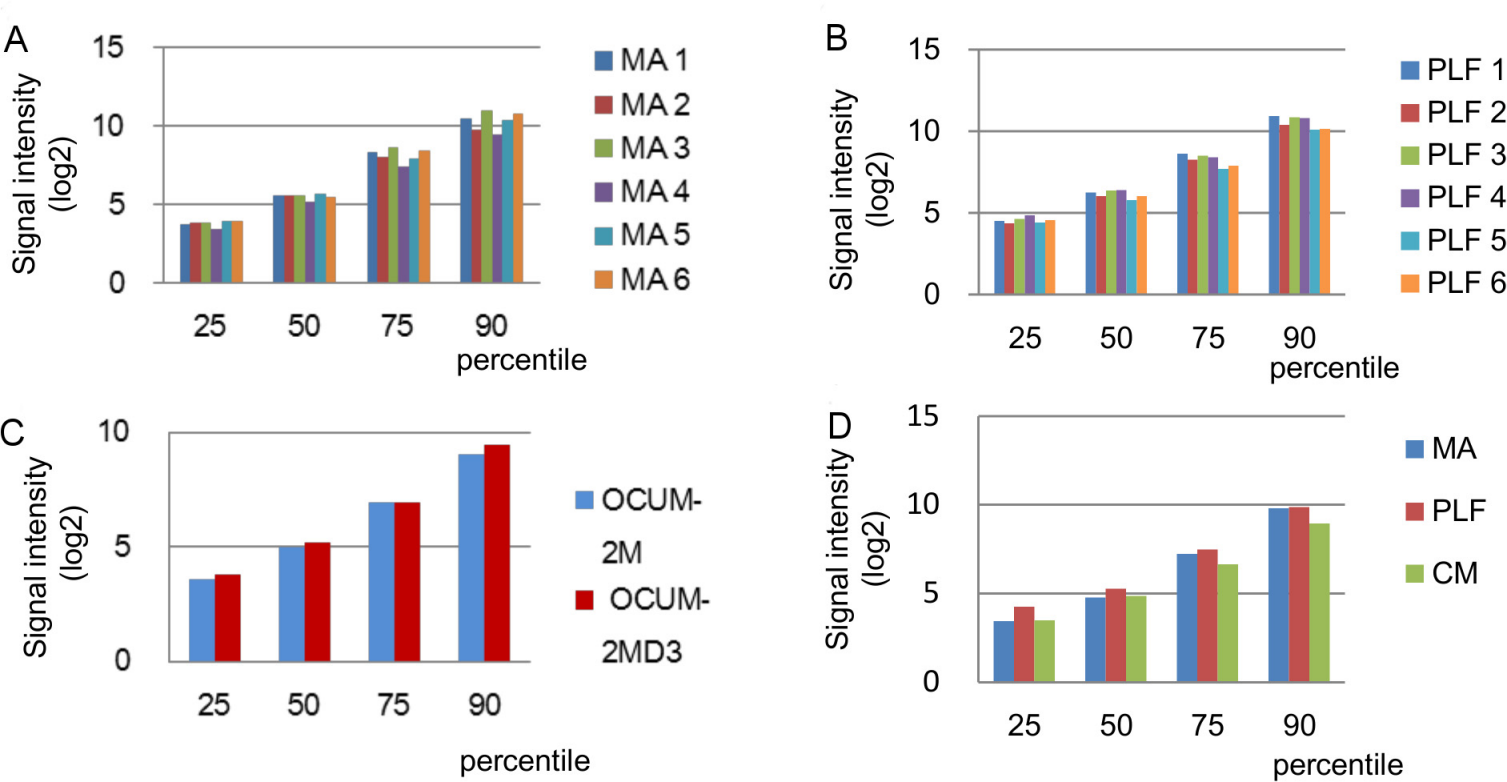
Signal intensity distribution among MA (A), PLF (B), and CM (C) samples and among the groups (D).

**Fig 4:**
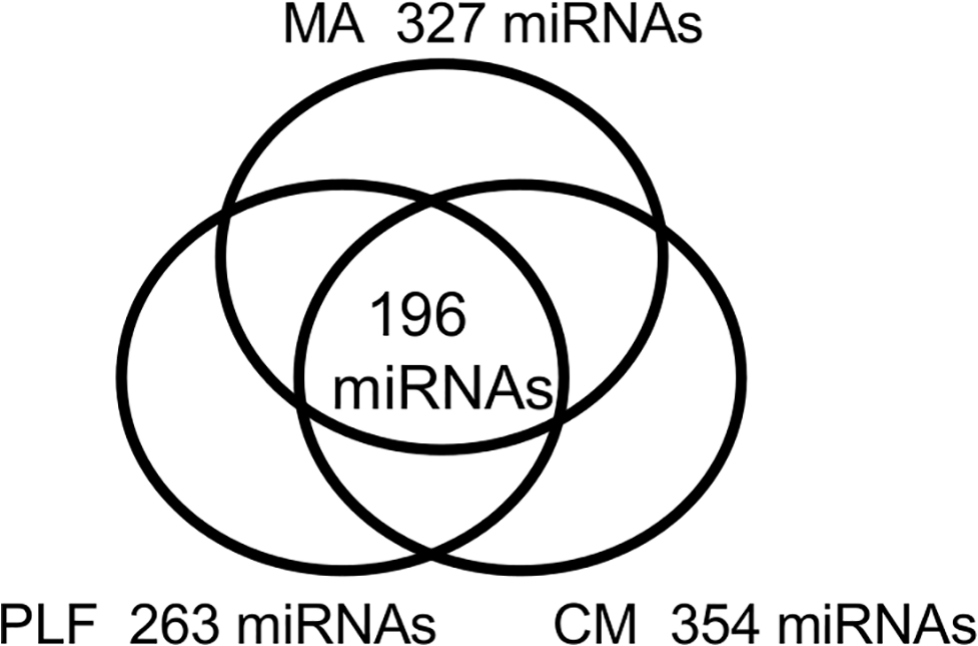
The number of miRNA species detected in malignant ascites, peritoneal lavage fluids, and cell culture media groups; 196 miRNAs were common to all groups.

Signal intensity distribution for each group is presented in Fig 3; this shows the processed signals normalised to the 25, 50, 75, and 90^th^ percentile in each group. In the 50^th^ percentile, the log_2_ relative signal intensities for MA (Figs 3–1) and PLF (Figs 3 and 2) samples and among three groups (MA, PLF, and CM, Figs 3 and 4) were 5.53 ± 0.19 (CV = 3.49%), 6.14 ± 0.24 (CV = 3.87%), and 4.97 ± SD (CV = 5.24%), respectively. The variability of signal intensity among the samples in each group as well as among the groups was low.

The numbers of miRNAs common among the samples in the MA, PLF, and CM groups were 327, 263, and 354, respectively, whereas this was 194 for the three groups (Fig 4).

Table 2 shows the correlation of miRNA expression between every two samples. The highest and the lowest CC values were 0.94 and 0.68, respectively; the average CC of all samples was 0.79 (CV = 8.86%). In MA, PLF, and CM groups, the average CC values were 0.85, 0.88, and 0.90, respectively, and the variability in the expression profiles for each group was low.

**Table 2:** Coefficient of correlation between samples. MA, Malignant ascites; PLF, Peritoneal lavage fluid; CM, Cell culture medium

Sample Name	MA 1	MA 2	MA 3	MA 4	MA 5	MA 6	OCUM-2M	OCUM-2MD3	PLF 1	PLF 2	PLF 3	PLF 4	PLF 5	PLF 6
MA 1		**0.78**	**0.8**	**0.79**	**0.82**	**0.83**	**0.78**	**0.78**	**0.72**	**0.69**	**0.7**	**0.73**	**0.7**	**0.67**
MA 2	**0.78**		**0.88**	**0.83**	**0.9**	**0.86**	**0.76**	**0.74**	**0.83**	**0.75**	**0.79**	**0.8**	**0.76**	**0.73**
MA 3	**0.8**	**0.88**		**0.86**	**0.9**	**0.88**	**0.79**	**0.76**	**0.79**	**0.69**	**0.72**	**0.76**	**0.72**	**0.69**
MA 4	**0.79**	**0.83**	**0.86**		**0.87**	**0.85**	**0.81**	**0.81**	**0.77**	**0.71**	**0.72**	**0.77**	**0.73**	**0.72**
MA 5	**0.82**	**0.9**	**0.9**	**0.87**		**0.91**	**0.81**	**0.79**	**0.81**	**0.73**	**0.77**	**0.77**	**0.75**	**0.71**
MA 6	**0.83**	**0.86**	**0.88**	**0.85**	**0.91**		**0.81**	**0.82**	**0.77**	**0.72**	**0.75**	**0.78**	**0.75**	**0.73**
OCUM-2M	**0.78**	**0.76**	**0.79**	**0.81**	**0.81**	**0.81**		**0.9**	**0.74**	**0.72**	**0.73**	**0.75**	**0.74**	**0.71**
OCUM-2MD3	**0.78**	**0.74**	**0.76**	**0.81**	**0.79**	**0.82**	**0.9**		**0.68**	**0.7**	**0.68**	**0.75**	**0.74**	**0.74**
PLF 1	**0.72**	**0.83**	**0.79**	**0.77**	**0.81**	**0.77**	**0.74**	**0.68**		**0.88**	**0.92**	**0.9**	**0.87**	**0.8**
PLF 2	**0.69**	**0.75**	**0.69**	**0.71**	**0.73**	**0.72**	**0.72**	**0.7**	**0.88**		**0.94**	**0.88**	**0.89**	**0.85**
PLF 3	**0.7**	**0.79**	**0.72**	**0.72**	**0.77**	**0.75**	**0.73**	**0.68**	**0.92**	**0.94**		**0.89**	**0.87**	**0.8**
PLF 4	**0.73**	**0.8**	**0.76**	**0.77**	**0.77**	**0.78**	**0.75**	**0.75**	**0.9**	**0.88**	**0.89**		**0.92**	**0.92**
PLF 5	**0.7**	**0.76**	**0.72**	**0.73**	**0.75**	**0.75**	**0.74**	**0.74**	**0.87**	**0.89**	**0.87**	**0.92**		**0.9**
PLF 6	**0.67**	**0.73**	**0.69**	**0.72**	**0.71**	**0.73**	**0.71**	**0.74**	**0.8**	**0.85**	**0.8**	**0.92**	**0.9**	

### Selection of candidate miRNAs related to peritoneal dissemination

**Fig 5:**
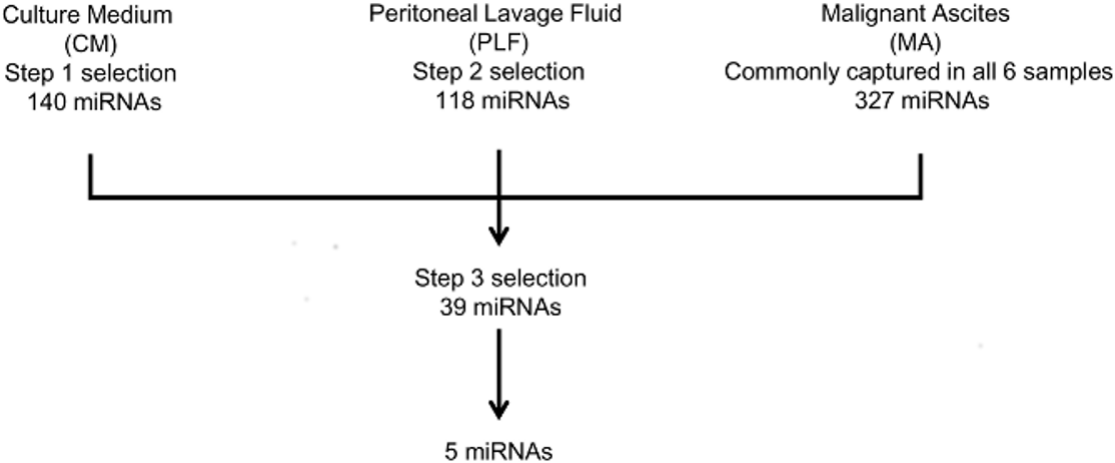
Identification of five exosomal miRNAs related to peritoneal dissemination. Schematic representation of the step-wise approach to screening miRNAs related to peritoneal dissemination. Step 1: selection of miRNAs differentially expressed (fold-change, >2) in culture medium (CM) of the highly metastatic peritoneal cell line OCUM-2MD3 (OCUM-2M parental cell line was used as control). Step 2: six peritoneal lavage fluid (PLF) samples were divided into T4 (n = 4) and T1–T3 groups according to gastrointestinal cancer stage; miRNAs differentially expressed (fold-change, >2) in the T4 group were selected. Step 3: the selected miRNA species common for Steps 1 and 2 and expressed in malignant ascites (MA) with signal intensity > log_2_ 10 in MA and PLF were considered as candidate miRNAs related to peritoneal metastases.

To date, no data have been available on the expression profiles of exosomal miRNAs associated with peritoneal dissemination, because of the unavailability of normal ascitic fluids against which to compare the MA expression profiles. We selected miRNAs related to peritoneal dissemination using a step-wise approach. The number of miRNAs commonly captured in the six MA samples was 327. In Steps 1 and 2, 140 and 118 metastasis-specific miRNAs were selected in the CM and PLF groups, respectively. From these 327, 140 and 118 miRNAs, five miRNAs were selected as candidates related to peritoneal dissemination (Fig 5). Two of them (miR-1225-5p and miR-320c) and miR-21 with the highest signal intensity among the MA-specific miRNAs were validated as being differentially expressed in 18 PLF samples, using qPCR (Table 3).

**Table 3:** Signal intensity and fold-change of the miRNAs. Malignant ascites (MA); Peritoneal lavage fluid (PLF); Condition medium (CM), T stage UICC 7^th^ edition (T4, T1-3); OCUM-2M (2M); OCUM-2MD3 (D3).

	Signal Intensity(log 2)		Fold-Change (log_2_)	
	MA	PLF (T4)	PLF (T4/T1-3)	CM (D3/2M)
miR-1225-5p	11.24	14.29	2.23	2.65
miR-320c	11.26	14.27	1.91	1.42
miR-1202	10.76	14.41	2.27	4.32
miR-1207-5p	10.94	14.23	1.9	1.52
miR-4270	11.10	13.71	2.25	4.70
miR-21	15.50	11.99	-0.55	-0.61

### Validation of candidate miRNA expression by qPCR

**Fig 6:**
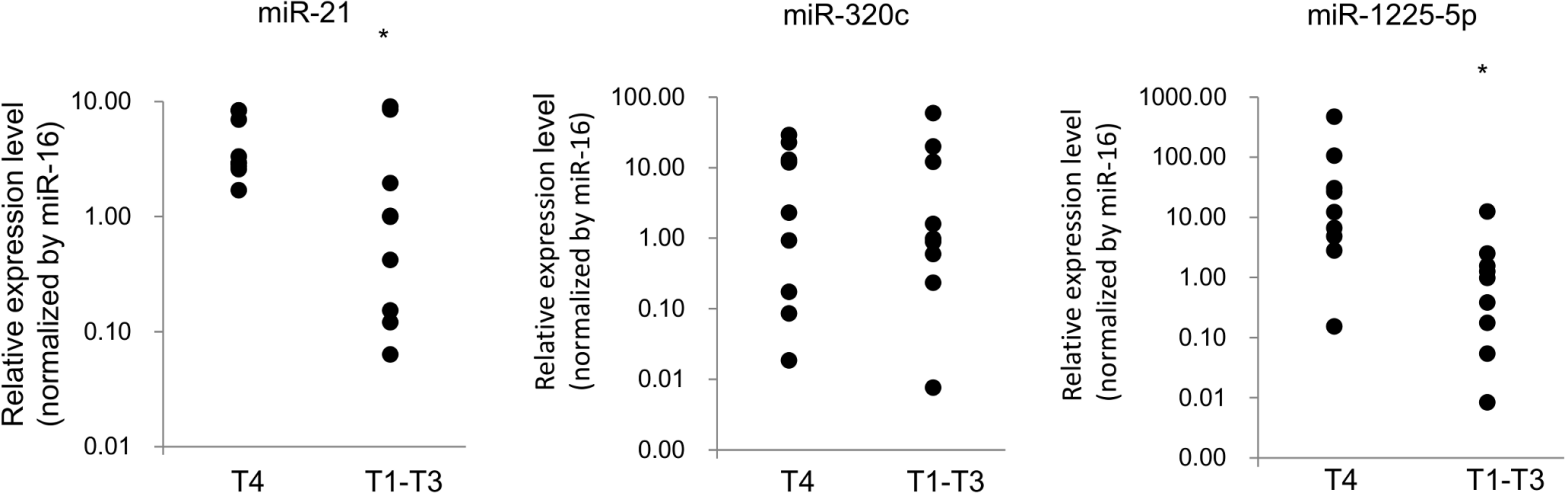
Relative expression levels of exosomal miRNAs in intraoperative peritoneal lavage fluid of T4 and T1–T3 gastric cancer patients. An asterisk indicates P < 0.05.

Eighteen PLF samples were divided into two groups according to the metastatic stage, T4 (perforated serosa, n = 9) and T1–T3 (subserosa or less, n = 9), and the expression of miR-21, miR-320c, and miR-1225-5p was analysed by qRT-PCR. Fig 5 shows that the levels of miR-21 and miR-1225-5p in T4-stage GC patients were significantly increased compared to those in T1–T3 patients (miR-21, P = 0.027, and miR-1225-5p, P = 0.008; Fig 5). The difference in miR-320c expression between two patient groups was non-significant (P = 0.928, Fig 6).

Next, the correlation between miRNA levels and clinical background of GC patients was examined (Table 4). MiR-21 and miR-1225-5p expression was significantly higher in patients with T4-stage cancer than that in T1–T3-stage patients (P = 0.015). No significant correlation was found between miRNA expression and other clinical parameters.

**Table 4:** Clinicopathological parameters and microRNA expression.

		miR-21			miR-302c			miR-1225-5p		
Variables	Cases	Low	High	P-value	Low	High	P-value	Low	High	P-value
Age(y)										
<70	11	5	6	1.000	6	5	1.000	5	6	1.000
≥70	7	3	4		3	4		3	4	
Gender										
Male	13	5	8	0.608	8	4	0.294	5	8	0.608
Female	5	3	2		8	5		3	2	
Histological grade										
Well and moderate^a^	7	3	3	1.000	4	3	1.000	3	4	1.000
Poor and other	11	7	5		5	6		5	6	
T stage										
T1-T2	9	7	2	0.015	5	4	1.000	7	2	0.015
T3-T4	9	1	8		4	5		1	8	
Lymph node metastasis										
Present	9	4	5	1.000	5	4	1.000	4	5	1.000
Absent	9	4	5		4	5		4	5	
TMN stage										
0–II	12	7	5	0.152	7	5	0.620	7	5	0.152
III–IV	6	1	5		2	4		1	5	

^a^Well-differentiated adenocarcinoma (well), moderately differentiated adenocarcinoma (moderate), poorly differentiated adenocarcinoma (poor), other histological type (other)

## Discussion

In this study, we investigated, for the first time, the miRNA content of exosomes isolated from MA and PLF of GC patients. Our study shows that exosomal miRNAs can be consistently extracted from MA and PLF and that miRNA expression profiles can indicate the status of peritoneum in GC patients.

The prognosis of GC with serosal invasion remains poor even after R0 resection, because of the high rate of recurrence, particularly peritoneal metastases (PM) [18]. PM in GC is the most complicated type of recurrence, because it is difficult to predict as well as to diagnose. Although ascites is the most common PM symptom, many GC patients with PM do not develop ascites. Among the imaging-based diagnostic methods, high-speed spiral CT is widely used for the assessment of preoperative staging of GC and post-therapeutic follow-up; however, in the case of PM its diagnostic accuracy is inadequate, mostly because of low sensitivity [19]. Using PET-CT for PM diagnosis in GC is also controversial, as it has been reported that FDG-PET has poor sensitivity for detection of PM in GC [20]. PLF has become the next target for the diagnosis and prediction of PM in GC. Cytological examination of intraoperatively collected PLF is used for the prediction of peritoneal recurrence [21] and is used for GC staging in Japan [22]. However, some investigators have reported that PLF cytology does not exhibit the sensitivity required for the prediction and detection of PM [21], and have proposed the use of molecular diagnostic methods, such as RT-PCR, for the detection of micro-metastases in PLF. In a multicentre prospective study, mRNA expression of the genes encoding carcinoembryonic antigen (CEA) and cytokeratin 20 (CK-20), evaluated by RT-PCR, has proven to be useful for the prediction of overall survival and PM in GC [23]. However, the disadvantage of mRNA-based diagnostic methods is the high degradability of mRNA in the course of surgical procedures.

In contrast, miRNAs enclosed in exosomes remain stable and can circulate in body fluids, such as serum, plasma, saliva, urine, breast milk, and tears, for long periods of time [24, 25]; exosomes have also been isolated from MA in ovarian cancer [26].

To the best of our knowledge, there has been no report on exosomal miRNAs isolated from the PLF of GC patients. Levänen et al. collected exosomes from bronchoalveolar lavage fluid and analysed exosomal miRNA expression to detect allergy-related miRNA species [27]. Liu et al. reported that exosomes isolated from cervicovaginal lavage fluid contained high levels of miR-21, which could be used as a biomarker of cervical cancer [28]. Here, we analysed the expression profiles of exosomal miRNAs in the PLF of GC patients by miRNA microarray technology in order to identify candidate diagnostic miRNA biomarkers for the prediction of metastasis in GC.

In our investigation, the first challenge lay in the quantitative isolation of exosomal miRNA from PLF. Weber et al. evaluated miRNA expression in 12 body fluids and reported that the total RNA concentration in peritoneal fluid was 775 μg/L [25], which was less than that we obtained from MA and PLF—4,400 and 6,400 μg/L, respectively The reason for the difference may be the methodology used for the isolation of total RNA, because Weber et al. directly extracted RNA from peritoneal fluid, whereas we first isolated exosomes and then used these for RNA extraction. Analysis of the quality of RNA isolated from PLF demonstrated an abundance of small (<200 nt) RNA species in CM and MA. There were no distinct 18S and 28S ribosomal RNA peaks in the preparation, indicating the absence of contamination with intracellular RNA.

The second problem encountered in the present study involved consistency in the quality of the exosomal miRNAs isolated. In our study, we observed low variability in the number of detected miRNA species and miRNA signal intensity, and a high correlation of miRNA expression among the samples in each group. The mean numbers of detectable miRNAs in MA and PLF were 490 and 367, which was in agreement with the number of miRNAs (397) previously detected in peritoneal fluid [25].

PLF and MA samples showed low variability in signal intensity (CV = 3.87% for the 50^th^ percentile). Furthermore, miRNA expression patterns were highly correlated between samples within each group (CC > 0.850), as well as between groups (0.8 > CC > 0.7). These results indicated that PLF could be a high-yield source of good quality miRNAs, which show consistent expression patterns in the microarray assay.

The miRNA expression profiles were analysed to select miRNAs specific for peritoneal metastasis. MiR-21 showed higher expression in patients with T4-stage carcinoma than in those of the T1–T3 group. Fabbri et al. reported that miR-21 and miR-29a in cancer-released exosomes affected tumour growth and spread by binding to and activating TLRs in the surrounding immune cells and inducing a prometastatic inflammatory response [29]; this supported the notion that tumour-derived exosomes contribute to the premetastatic microenvironment [30, 31]. This may indicate that T4 carcinoma-derived exosomes create the premetastatic niche in the peritoneum, which then supports peritoneal invasion after curative resection of GC.

Using a step-wise approach, we selected five exosomal miRNAs (miR-320c, miR-1202, miR-1225-5p, miR-1207-5p, and miR-7270) and validated miR-320 and miR1225-5p expression in the PLF of 18 CG patients by qRT-PCR. Mir-1225-5p showed higher expression in T4 than in T1–T3 stage CG patients, confirming the results obtained by microarray, whereas there was no difference in miR-320 levels between patient groups. Mir-1225 targets the polycystic kidney disease gene, *PKD1*, which is frequently mutated in autosomal dominant polycystic kidney disease; thus, miR1225 may affect the progression of this disease [31]. However, the relationship between miR-1225 and cancer remains unclear. MiR-21 and miR-1225-5p may prepare a premetastatic niche in the peritoneum for the dissemination and settlement of metastasising cancer cells and thus may indicate the predisposition for the peritoneal recurrence after curative GC resection.

PLF yields information about the status of the peritoneum, such as changes in the population of immune cells and secretion of growth factors and cytokines [32, 33]. Cytology and molecular diagnostic assays are based on the detection of cancer cells, whereas profiling of miRNAs in PLF may be used for the prediction of a peritoneal premetastatic phenotype in GC, ensuring more effective preventive and curative measures.
